# Lean adoption in hospitals: the role of contextual factors and introduction strategy

**DOI:** 10.1186/s12913-021-06885-4

**Published:** 2021-08-28

**Authors:** Angelo Rosa, Giuliano Marolla, Federico Lega, Francesco Manfredi

**Affiliations:** 1 Department of Management Studies, LUM University, S.S. 100 Km, 70010 Casamassima, Italy; 2 Center in Health Administration, and Center for Applied Health Economics and Management of IRCCS Galeazzi, University of Milan, Via Giacomo Venezian, 1, 20133 Milan, Italy

**Keywords:** Lean implementation, Contextual factors, Introduction strategy, MUSIQ, Case study

## Abstract

**Background:**

In the scientific literature, many studies describe the application of lean methodology in the hospital setting. Most of the articles focus on the results rather than on the approach adopted to introduce the lean methodology. In the absence of a clear view of the context and the introduction strategy, the first steps of the implementation process can take on an empirical, trial and error profile. Such implementation is time-consuming and resource-intensive and affects the adoption of the model at the organizational level. This research aims to outline the role contextual factors and introduction strategy play in supporting the operators introducing lean methodology in a hospital setting.

**Methodology:**

The methodology is revealed in a case study of an important hospital in Southern Italy, where lean has been successfully introduced through a pilot project in the pathway of cancer patients. The originality of the research is seen in the detailed description of the contextual elements and the introduction strategy.

**Results:**

The results show significant process improvements and highlight the spontaneous dissemination of the culture of change in the organization and the streamlined adoption at the micro level.

**Conclusion:**

The case study shows the importance of the lean introduction strategy and contextual factors for successful lean implementation. Furthermore, it shows how both factors influence each other, underlining the dynamism of the organizational system.

**Supplementary Information:**

The online version contains supplementary material available at 10.1186/s12913-021-06885-4.

## Background

Over the last decade, healthcare has been called upon to respond to the increasing pressures arising from changes in demand – due to epidemiological changes and the demand for quality and safety – and increased costs due to the introduction of new technologies [1, 2]. These major challenges are exacerbated by the shrinking resources available in health systems and, for most countries, by the principle of universal access to patient care. In order to meet the patients’ needs, a hospital must utilize a number of scarce resources at the right time: beds, technological equipment, staff with appropriate clinical skills, medical devices, diagnostic reports, etc. [1, 2].

One of the most relevant issues for the management of a healthcare provider is the management of patient flows in order to purchase, make available, and use these scarce resources at the right time and in the right way, and to ensure the best possible care [3–5]. In this scenario, hospitals need to focus on the patient pathways in order to ensure fast, safe, and high-quality service [3, 6–8]. The search for solutions to these challenges has extended beyond the boundaries of healthcare practices to study organizational methods and paradigms that have been successfully implemented in other sectors [3, 5]. Among these, lean thinking has proven to be one of the most effective solutions for improving operational performance and process efficiency and for reducing waste [5, 9]. Lean is a process-based methodology focused on improving processes to achieve a customer ideal state and the elimination of waste [10]. Waste is defined as the results of unnecessary or wrong tasks, actions or process steps that do not directly benefit the patient. The taxonomy of waste is: overproduction, defects, waiting, transportation, inventory, motion, extra-processing and unused talent [3–5]. In addition, lean addresses other key service issues such as continuous improvement and employee empowerment, whether healthcare professionals or managers [1, 11, 12]. Lean healthcare is defined as a strategic approach to increasing the reliability and stability of healthcare processes [7, 13, 14].

The first documented cases of lean applications in a hospital setting (HS) date back to the late 1990s. These aimed at improving patient care processes, interdepartmental interaction, and employee satisfaction [1, 2]. The Virginia Mason Medical Center is one of the first and most emblematic examples of a successful migration of lean methodology from the manufacturing sector to healthcare. The hospital, based on the principles of the Toyota Production System, created the Virginia Mason Production System, a holistic management model in continuous evolution that not only had a strong impact on the quality of the services provided and on the reduction of lead time, but it also led to a decrease in operating costs [14, 15]. Over time, many hospitals have followed in the footsteps of the Virginia Mason Medical Center [8, 16, 17]. The lean paradigm crossed the US border and spread to other countries such as Canada and England [5, 12]. It was not until the early 2000 that the model was introduced in European hospitals [12, 16].

The implementation of the lean paradigm in HS environments has increasingly attracted the attention of researchers and professionals. The interest in lean in HSs was fostered by the idea that the paradigm was particularly suitable for hospitals because its concepts are intuitive, compelling, and, therefore, easy for medical staff to use [18, 19]. However, over time, alongside the evidence of successful implementation of lean in HSs, much of the research has shown failures in adopting the paradigm [5, 20, 21]. Moreover, a literature review showed that most of the cases were characterized by a partial implementation of lean methodologies and concerned single processes in the value chain or restricted technical applications [20, 22]. Even today, few hospitals apply lean principles at a systemic level [23, 24].

The failure of lean implementation is a hot topic. Many authors who have focused their studies on social and managerial issues have highlighted the existence of factors that either enable or hinder the implementation of lean. These factors are mostly related to the context and the implementation strategies [5, 16, 25–27]. Lean implementation is not self-evident, and the process of transforming an organization into a lean organization requires a long-term strategic vision, a commitment by management, and a culture of change in the entire organization [5, 16, 26]. Contextual factors influence successful implementation and introduction strategy; lean adoption, in turn, changes contextual factors. A lean transformation must be planned and managed; it is not a quick solution, but a strategic plan in constant evolution [5, 28, 29]. From this point of view, the introduction phase plays a fundamental role in implementation because it facilitates the dissemination of the lean principle in hospitals and enables the contextual elements that support change. Although most researchers have recognized the role of the introduction step, the impact of this phase on contextual factors has been poorly reported on in the literature [5, 12, 20]. Most of the articles have focused more on the benefits of this phase than on how to manage it.

In light of this, it is necessary to examine how hospitals introduce lean into their clinical pathways in order to explain the success of the lean implementation. Starting with an in-depth analysis of the contextual factors discussed in the literature, the document helps to clarify what drives success in lean implementation within the hospital. The research has therefore undertaken a critical study of the introduction of lean in the case study of the haematology ward at a university hospital in the south of Italy. The objective is to highlight: (a) the role of contextual factors for successful lean introduction and implementation in a hospital ward; (b) how the pilot project has improved the pathway of a cancer patient undergoing chemotherapy infusion; and, (c) how the success of the pilot project modified the contextual factors, facilitating the spread of lean within the organization.

The study has the merit of detailing all the lean introduction phases. The analysis period is about 2 years. The lean introduction started in May 2018 and lasted 7 months. The pilot project results refer to the follow-up period of December 2018 to May 2020, while the dissemination results refer to the period from December 2019 to May 2020.

The paper is structured as follows: In the following section, the theoretical background is provided. Section 3 describes the research methods, while Section 4 presents the results of the pilot project. Finally, Section 5 presents the conclusion, highlights some limitations of this study, and proposes some directions for further research.

### Theoretical background

Most authors point out that the introduction phase is a crucial moment in lean implementation [10, 12, 16]. This phase reduces distrust of the method and organizational resistance to change. It shows the benefits of lean and assesses the organization’s ability to undertake continuous improvement. Many case studies report the success of lean in HSs by describing the use of lean instruments [8, 30, 31]. They offer the practitioners some methodological support, but not in a structured way since they do not provide a clear implementation roadmap [5, 32, 33]. Some authors have tried to fill this gap in the literature by offering guidelines for implementation. Augusto and Tortorella [33] suggests carrying out a feasibility study focused on the desired performance before implementing continuous improvement activities. The author suggests defining the techniques, roles, and results related to the improvement path. Curatolo et al. [5] argue that the improvement procedure has to take into account six core operational activities of business process improvement and five support activities. The six core operational activities are: selecting projects, understanding process flows, measuring process performance, process analysis, process improvement, and implementing of lean solutions. The five support activities are: monitoring, managing change, organizing a project team, establishing top management support, and understanding the environment. These studies, while offering further guidance on the process of introducing lean into a hospital, do not describe either the organizational context in which the method is being implemented or the strategies for its implementation [5, 12, 25]. The introduction of lean into a HS is not an easy task; there are many organizational issues to be addressed. Among these, the analysis of the context and the definition of the implementation strategy are the ones with the greatest impact on the success of the introduction [16, 26, 34].

The contextual elements are the special organizational characteristics that must be considered to understand how a set of interventions may play out [35, 36]. They interact and influence the intervention and its effectiveness [34, 36]. Two of the most cited contextual element are the drive to improve processes and the level of maturity [5, 10]. The drive for improvement is represented by the exogenous and endogenous needs that act as triggers for the introduction of improvement methodologies [25, 26, 35, 37]. The level of maturity refers to knowledge and experience in process improvement initiatives. It includes knowledge of methodologies and tools, experience gained, confidence, trust, and dissemination within the organization. Where the maturity is low, there is a risk of lean introduction failure in both the processes and the organization as a whole [5, 16, 38]. As long as the organization does not reach a fair level of maturity, the rate of change tends to be slow and sometimes frustrating. However, as the degree of maturity increases, lean implementation becomes a “day-to-day job” rather than a series of projects that take place at discreet moments [10, 21, 39]. Hasle et al. [39] highlighted that a high level of maturity allows for the implementation of principle-driven lean. Contextual elements include organizational and technological barriers such as resistance to change, lack of motivation, skepticism, and a lack of time and resources that inhibits the introduction and the implementation process [4, 8, 21, 40]. The lean introduction process in HS is also complicated by the organizational context and the double line of clinical and management authority in hospitals [41, 42].

With regard to internal contextual factors, many authors explored the readiness and sustainability factors influencing the adoption of lean. Readiness factors are those elements that improve the chances of lean implementation success; they provide the necessary skills and knowledge to enable organizational change [23, 43–45]. The readiness and sustainability factors include any practices or characteristics that allow organizational transformation by reducing or nullifying potential inhibitors of success. High commitment and strong leadership of managers and physicians, continuous training, value flow orientation, and the hospital’s involvement in continuous improvement are just some of the most discussed topics [5, 10, 16, 43]. Other examples include understanding employees needs, identifying the organization’s strategic objectives, project management, and teamwork [5, 12, 16, 46].

From the study of the contextual elements described so far, some authors have developed models to assess the impact of context on the implementation of organizational improvement activities. Kaplan et al. [36] put forth the Model for Understanding Success in Quality (MUSIQ). The authors identified 25 key contextual factors at different organizational levels that influence the success of quality improvement efforts. They defined five domains: the microsystem, the quality improvement team, quality improvement support and capacity, organization, and the external environment. Kaplan et al. [36] suggest that an organization that disregards contextual factors is doomed to fail in implementing an improvement program; an organization that adopts a context-appropriate implementation strategy can change the outcome by triggering implementation enablers. Previous studies of lean adoption in HSs suggest that the fit between the approach taken and the circumstances will influence the chances of success [3, 12, 34].

There are two strategies for introducing lean in a HS, and they are characterized by the implementation level. The level of implementation refers to either micro or meso implementation. Brandao de Souza [16] defined meso-level implementation as the condition under which lean is spread throughout the organization and is implemented at the strategic level, while micro-level implementation is where lean is implemented at a single process level in discrete moments. Meso-level implementation is crucial for long-term success because a lack of integration in a lean system can lead to the achievement of local rather than global objectives and can also affect the sustainability of the paradigm [23, 26, 47]. However, organizations that want to implement lean at the strategic level often do not recognize the need for a long-term implementation program and introduce lean as a “big-bang initiative”. This leads in many cases to a failure to introduce the method [16, 47]. Many researchers suggest introducing the lean approach through a pilot project run by a specially formed lean team [12, 16, 48, 49]. The pilot project should be challenging, involve a process relevant to the organization, and require the use of a systemic approach. In particular, it should not be limited to the application of “pockets of good practice” or lean tools, but should include the systemic adoption of improvement programs such as the Plan-Do-Check-Act (PDCA) cycle [21, 48]. Brandao de Souza [16] asserts that the first initiative should be tested on a relevant patient pathway. The lean team should be composed of clinical and non-clinical staff actively involved in the patient pathway. A pilot project that meets these conditions is a useful tool for increasing the maturity of the method within the organization [21, 39]. It can increase the confidence of the team and staff in the lean approach and can promote the learning of lean methodologies and techniques [21, 39]. Moreover, the pilot project activates the contextual elements, enabling the introduction of the model [10, 12]. The successes of the pilot initiative must be celebrated and communicated within the organization [10]. When the first initiative leads to visible and easily quantifiable results, the method has a greater chance of spreading throughout the organization [10, 12, 16]. In light of these considerations, the lean implementation requires that the contextual elements and the introduction strategy be assessed at the same time. In addition, it would seem fair to assume that as contextual factors influence the introduction strategy, the results of the implementation strategy will influence the contextual factors.

**Fig. 1:**
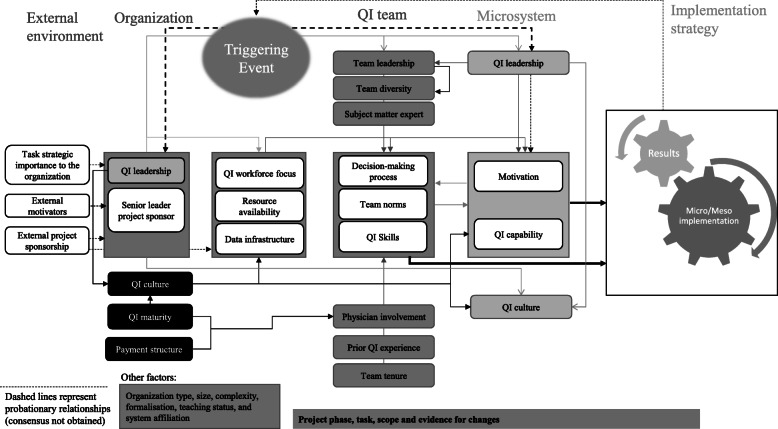
Our adaptation of the MUSIQ model

In Fig. 1, we propose an adaptation of the MUSIQ model [36] that shows the impact that the lean implementation strategy has on the contextual elements.

## Methods

### Study setting and design

This is an explanatory single-case study of the introduction of lean at a university hospital in Southern Italy. In particular, the introduction of lean in the pathway of a cancer patient undergoing infusion chemotherapy in a haematology ward will be discussed. This study was designed to evaluate how the contextual elements discussed so far have influenced the introduction of the method and how the successful pilot project has enhanced the internal context. We used the adaptation of the MUSIQ model [36] proposed in Fig. 1 to systematically trace the antecedents of the lean introduction and to explain how the success of the implementation strategy changes the contextual elements.

**Fig. 2:**
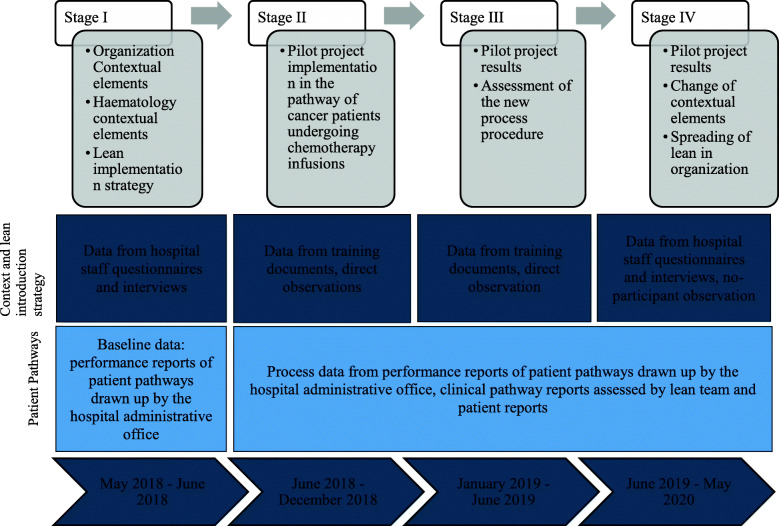
Stages of data analysis

The work covers four periods over 2 years (Fig. 2). The first period concerns lean introduction and implementation strategy. The second is related to the pilot project implementation in the haematology ward. The third shows the pilot project results. The last assesses the impact of the pilot project on the dissemination of lean within the organization.

### Data collection

Different data sources and data collection methods are used with the aim of improving data validity through triangulation. The data sources are lean training documents, direct observations and nonparticipant observations, process performance reports, process data recorded by patients, and two questionnaires submitted to the hospital staff (the questionnaires assess the “pre” and “post” lean dissemination phases and the difference regards three open questions) (Fig. 2). The second author is the consultant who trained the lean team and coordinated the pilot project, and the first author conducted approximately 50 h of nonparticipant observations. The questionnaire was delivered to 25 medical department staff members in September 2018 and in May 2020. The first questionnaire focused on contextual factors that existed before the introduction of lean, and the second investigated changes in the contextual elements - in particular trust, maturity and lean dissemination. The semi-annual performance reports from 2017 to 2020 for the clinical pathway under examination include daily averages of the number of chemotherapies per chemo chair (MT), the patients’ length of stay (LOS), and the daily average of the percentage of patients undergoing chemotherapeutic infusion within 3 hours of hospital admission (P3). Each day, from September 2018, a document containing all the steps of the clinical pathway was given to each patient. For each activity, the patient recorded the start and end time, and a signature of the doctor or nurse was required. In the period of September 2018 – May 2020, the medical staff collected more than 1.250 reports from patients. The study also draws on 10 semi-structured interviews. The hospital CEO, the chief of the medical department, the nurse supervisor, the chief of the antiblastic chemotherapy handling units, and the chief of the clinical laboratory were interviewed in September 2018 and May 2020. The interviews focused on the contextual elements either enabling or inhibiting lean introduction or its dissemination, and ranged from 30 min to 1 hour in duration.

### Data analysis

The factors described in Fig. 1 were used to systematically analyse the antecedents of the results and to understand their causal influence on the lean introduction. This data collection allows for the description of the case study. In addition, it simplifies the interpretation of the evidence that emerged through the study of the factors listed. The authors carried out a content analysis to classify the data by theme. The content analysis followed an inductive approach based on the identification of meaning units at the semantic level and the encoding of results [49, 50]. Whenever researchers did not agree on semantic meaning, a new unit of analysis was proposed. The principle of consensus among all panel members was used to determine the interpretation, addition or deletion of elements of analysis. The discussion of the case study focuses on four themes: (a) contextual elements enabling or hindering lean introduction, (b) implementation strategy, (c) pilot project results, and (d) lean dissemination and adoption in hospital. These themes were submitted for review by the interviewees; their feedback was used to improve the accuracy of the case study description.

## Results

### Case study presentation

The university hospital is a model of excellence in Italy for pre-clinical, translational, and clinical research and care activities. It is equipped with 110 beds to treat all types of oncological pathologies in adults. There are 115 researchers working there. The hospital is structured into six departments, of which three are clinical (Medical Area, Diagnosis and Imaging Therapy, Surgical Area), two are services, and one is an administrative/management department. The medical area includes four wards: medical oncology for thoracic pathology, medical oncology, haematology, medical oncology for oncology patient care. In 2015, the institute was accredited as a clinical cancer centre according to the Organization of European Cancer Institutes (OECI). Since 2015, evidence-based medicine and patient-centred care methodologies have been successfully implemented in the hospital, but no process improvement methodology has been used. In 2017, the hospital became a hub for oncological diseases, which led to an increased demand for care and services. The hospital has received national funds dedicated to hubs and has made investments in infrastructure improvements and the purchase of new innovative medical equipment.

#### Contextual factors enabling or hindering lean introduction

The description of the external and internal contextual factors, as revealed in the first questionnaire and the interviews, is given in Table 1. Below is a brief description of each item.

**Table 1:**
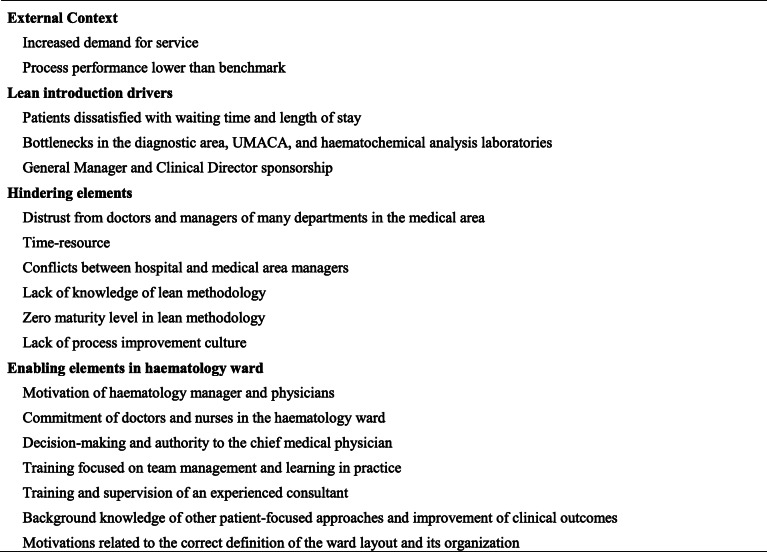
External and internal contextual factors recognized by hospital staff

### External context and organizational elements driving lean introduction in the haematology ward

The analysis of the context revealed external and internal elements influencing the introduction of lean. Starting with the external elements, the most frequently discussed motivators that led to the search for methodologies for process improvement include the continuous increase in patient volume and the benchmark of process performance with other providers. Although the clinical results were above the national average, the increase in demand - especially in the medical area - highlighted the inability to manage the increasing flow of patients. The inability to manage the increasing number of patients also affected the performance of the process in the diagnostic area.

Internal elements driving the lean introduction were related to dissatisfaction with inefficient work practices within the medical area and the dissatisfaction of many patients who complained about long wait times and lengths of stay.

The choice of lean methodology derives from the desire to follow the example of certain Tuscan hospitals that have been using lean at a strategic level since 2015. These hospitals are considered the benchmark for continuous process improvement. One of these hospitals was already included in the 2013 OASI Report, edited by CERGAS - Bocconi, among the six Italian companies that were the first and best to successfully implement Lean Thinking in healthcare. In addition, the methodology was strongly sponsored by the clinical director and the general director of the hospital. They had participated in a 60-h regional training course on lean healthcare in the second half of 2017. During the training course, they studied case studies of excellence in lean implementation.

When, in May 2018, the hospital directorate proposed the introduction of lean methodology in the medical area, the head physicians showed strong resistance because of the resources that would need to be allocated to the implementation process. In addition, some doctors did not trust the method. This brought up some conflicts with the medical area managers. The haematology staff, represented by their head physician, were the only ones who explicitly agreed to implement the lean introduction. The department, as in most Italian hospitals, is structured as a clinical area where the physicians - in contrast to other professionals - were members of the ward organizationally. Haematology staff were strongly motivated to do research and achieve excellent process performance. They were interested in taking the opportunity to define excellent clinical pathways, as the ward was undergoing managerial and layout restructuring. In addition, the haematology staff believed that lean could further improve clinical performance and improve the patient-centred and evidence-based approach. Until mid-2017 the ward was part of oncology; afterward, it was made independent and new areas of the hospital were assigned to it. Since the ward became independent, one head physician, three doctors and four nurses have been hired. The department is equipped with the most modern medical equipment. The layout of the ward was not yet fully defined, and some rooms that could have potentially been assigned to medical, diagnostic and therapeutic activities had not been assigned to process activities. The ward shares the Antiblastic Chemotherapy Handling Unit (UMACA) and the analysis laboratory with the other four medical department wards in the hospital, so the staff needed to coordinate clinical processes so as not to create bottlenecks.

Since haematology is a strategic ward for the hospital, and in the last 2 years the demand for treatment has increased more than in other wards, the managers of the medical area have deemed it appropriate to introduce lean there. Haematology ward is considered strategic due to its high attractiveness and high immigration rates of patients from outside the region. These phenomena derive from the excellent reputation of the department in relation to the quality of care. Although the clinical pathways were characterized by excellent clinical outcomes, qualitative benchmarking activities (based on testimonials from physicians and patients) showed that the organization of the haematology patient pathway was very different compared to the benchmark (a Tuscan hospital) and that the patients’ perception of non-clinical service quality was lower. Although no investigation was carried out with respect to the ratio of equipment and personnel available per number of patients and amount of activities regarding the hospitals taken as benchmarks, the testimonials prompted management to come up with new specific, measurable, attainable, relevant and time - bound (SMART) goals (Table 2). The goals will be described in the next section.

**Table 2:**
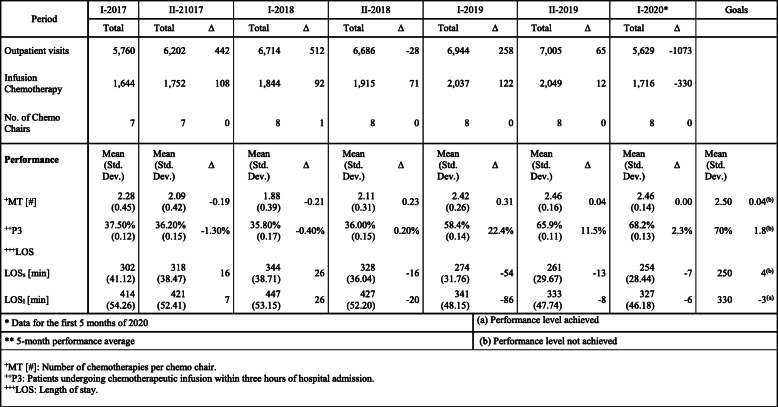
Performance indicators of cancer patient pathways in the haematology ward with semi-354 annual variation and benchmark

### Internal contextual elements enabling and hindering lean introduction in the haematology ward

At the organizational level, hospital management has strongly supported the introduction of the method. Since the haematology staff had no experience in process improvement activities, management provided the budget for an external consultant. In addition, three non-clinical personnel were allocated part-time to support the implementation of visual management systems and communication. The organizational structure of the ward has been modified to a matrix form. A team of three haematology ward physicians and two nurses was established and the ward’s head physician was elected project manager. The project manager had formal authority over the team and the personnel employed in the process to be improved; this reduced conflicts due to the double line of hierarchical authority. In this phase, the top-down decision-making approach was crucial to the successful restructuring of the organizational structure and the definition of the new organizational roles. The lean advisor supported the group for 8 months through training and project supervision. He coordinated two meetings per week and carried out Kata coaching activities. The theoretical training activity, lasting 5 week ends (in June 2018), was differentiated to accommodate technical and managerial competency needs. The team project manager and the medical area manager were trained on topics such as project management, team management, leadership, and the dissemination of lean. The members of the lean group were trained in lean techniques and tools. The key principles of lean thinking, the PDCA cycle methodology and lean assessment were taught to all participants. The most difficult barrier to overcome was the time available. The team agreed to spend 8 hours per week on training and pilot project implementation. The management of the team was facilitated by the experience gained with the implementation of the patient-centred care and evidence-based medicine. The motivation of the medical staff–microsystem element–and the focus on team management were key success factors for the involvement of team members. The culture of change introduced by patient-centred and evidence-based medicine was another enabling factor.

#### Implementation strategies

##### Pilot project definition

Hospital managers and lean team members, who had experience in implementing patient-centred care methodologies, suggested starting a pilot project for the lean introduction. The consultant agreed. The team, with the support of the expert, analysed the clinical pathways in haematology. Six pathways emerged: a) diagnostic visits, b) biopsies, c) check-up visits, d) transfusions, e) infusion chemotherapies, and f) oral chemotherapies. Hospital managers argued that the pathway of the patient undergoing infusion chemotherapy was the most critical for patient and organization value. This process is the only one that involves several departments and requires a large amount of materials and time-consuming resources. In the first and second half of 2017 and 2018, there was a significant increase in the number of chemotherapeutic preparations. LOS, P3, and MT performance decreased during the same periods (Table 2). In addition, outpatient visits and the number of biopsies also increased. The medical staff stated that the increase in demand in the medical area had particularly affected the infusion therapy activities because they involved technical and instrumental resources that are shared with other departments (Table 2). The length of stay was analysed for patients undergoing short (LOS_s_) and long-term infusion (LOS_l_) chemotherapy. The first has a minimum duration of 90 min and a maximum of 180 min, and the second has a minimum duration of 181 min and a maximum of 360 min. Each patient was assigned to one of the infusion treatment classes. Process data were collected and analysed by the Department Management Control Office. The process performance data collection and reports were established in 2015 for the implementation of evidence-based medicine.

##### Pilot project implementation

The pilot project started in June 2018. The first month was dedicated to Gemba Walk, Methods-Time Measurement (MTM) and implementation of the 5S. In addition, the consultant trained the project manager, department managers and lean team members. There were many difficulties during the training period, especially with regard to process mapping and the concept of value, the latter being interpreted by doctors as clinical output. The non-medical staff dedicated to the project assisted the team in the drawing of the visual management material. A room in the medical department was dedicated for team meetings, and some notice boards were installed to post the materials developed during the project. The project activities were organized according to the Report A3 scheme. It followed the phases of the consolidated Deming cycle: Plan-Do-Check-Act (PDCA). Implementing the approach proposed by Deming allowed for the trial-and-error empirical method to be abandoned in favour of the “scientific” one. The PDCA allowed accurate planning of objectives and activities and their monitoring. The departmental managers and the consultant through the study of the national publications and explicit requests to colleagues in other hospitals - considered virtuous - identified the benchmark (Table 2). They took into account the hospital’s specific characteristics, such as the policy of not accepting haematochemical reports from outside for fragile patients. This choice is dictated by the risk management plan and affects P3 and MT performance. Time for blood sampling and haematochemical analysis is added to the cycle time; however, it eliminates many risks associated with clinical treatment.

**Fig. 3:**
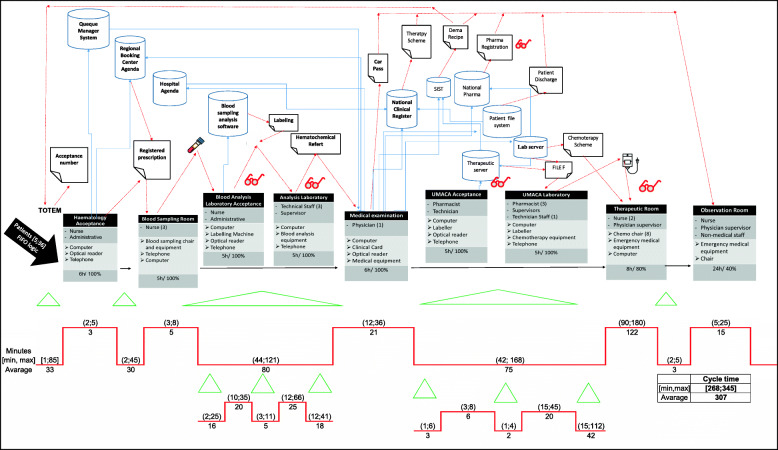
As-is process represented by VSM

The existing care process was mapped through Value Stream Mapping (VSM) based on the patient reports, Gemba walks, interviews, and direct observation. For instance, Fig. 3 shows the pathway of a patient undergoing short-term infusion chemotherapy. The cycle time in Fig. 3 was calculated over an observation period of 1 week and included 51 patients. In addition, the application of the Demand Map and the Spaghetti Chart were used to evaluate the ward nodes activated by the patients and the ward’s layout. These tools were useful in defining the possible sources of waste in the process. The application of these tools lasted more than 2 months and required several revisions. Once completed, the results were posted in the meeting room and were used for discussions with colleagues in the medical department. The lean team requested support from the consultant for the drafting of the VSM and for the layout analysis. In addition, the consultant was asked to simplify negotiations with staff from other departments who were reluctant to be subjected to time and method measurements. The negotiation activity required a degree of organizational effort. The facilitating elements were manifold: they enabled the involvement of staff opposed to the introduction of measuring instruments. In particular, the most effective were: the intervention of the directorate general, the delegation of hierarchical authority to the project manager and finally the endorsement of trade union committees. Moreover, during the planning phase, many difficulties emerged, including the selection of a unique and standardised measurement system, the coordination of work and meeting schedules, and the deadlines set by the project Gantt. Although the project manager was able to manage the team, he did not have enough experience in lean tools. The external consultant played a key role in managing these activities.

At the end of the as-is analysis process, an Ishikawa diagram was used for the definition of root causes. Four root causes emerged from the meetings and interviews. They were patient flow management, coordination activities with other departments, layout, and Information Technology equipment (IT).

Patient flow management concerned the absence of priority in the management of patients based on the clinical path and the arrival of patients in the early hours of the morning. The lack of coordination with other departments led to delays in the preparation of infusion chemotherapy and blood test reports. The layout was such that the flow of doctors and nurses crossed the flow of patients, and this caused great inconvenience to the doctors and nurses. Also, the computer software was not compatible, which meant that the same data had to be recorded several times.

After some meetings and rigorous brainstorming, the lean team suggested changes to be made in the existing pathway. This was done by considering how patients could be divided into clusters so that the infusion activity could start as soon as possible without affecting other occurrences. Moreover, it is made possible to simplify the coordination between diagnostic units. The patient flow has been managed in such a way that long-term patients are given priority (first to be accepted and blood sampled), followed by patients needing biopsies, first visit, follow-up visit. Finally, short-term patients are treated in a way that limits waiting time and does not affect the activities of other departments. Theories of queues and operational research methodologies were implemented to address chemo chair saturation. A chemo chair activities plan was implemented through pull logic. In addition, the hospital engineer was involved in making sure the information systems were compatible. Whenever integrating the software was not possible, a data entry person was assigned to prevent medical staff from wasting their time on low-value activities. The ward layout has been modified to prevent patient flows from intersecting with the flows of doctors and nurses. In addition, the use of one room has been changed from a small warehouse to a blood collection room to increase the value of the activities carried out within it. The waiting rooms were moved outside the ward and, during the first 2 hours of the working day, the biopsy room was reassigned to blood collection activities to speed up the requests for therapies in UMACA. Patient intake, blood collection, and tube labelling activities have been paralleled to be performed simultaneously in the same room. The routes and modalities for the delivery of blood samples to the laboratories were revised in order to reduce the time and distance travelled by non-clinical staff. Tablet reporting systems were installed. Finally, a patient chemo chair allocation system was developed.

The resources needed for these changes were determined. The team tested and modified the changes during December 2018 and January 2019. The tests were evaluated based on the performance data, patient reports and the team’s expertise.

#### Pilot project results

In January 2019, it was decided to implement the new standard procedures that were tested in order to improve performance. The team met once a week for 6 months. On a monthly basis, performance was reviewed and new changes were tested. Clinical and nonclinical personnel from other wards and departments were invited to each weekly meeting to share with them the results of the pilot project, and to involve them in the lean methodology.

Every morning, the team leader investigated the impact of organizational changes in order to avoid conflicts. Organizational problems that emerged were discussed and resolved by consensus. In the follow-up phase, the consultant performed supervisory activities. Each week, the team leader performed the Kata coaching. During the first 6 months, the monitoring of activity was very frequent to prevent a return to old operating modes. Subsequently, when the staff had learned the new procedures, monitoring was reduced to once a month.

**Fig. 4:**
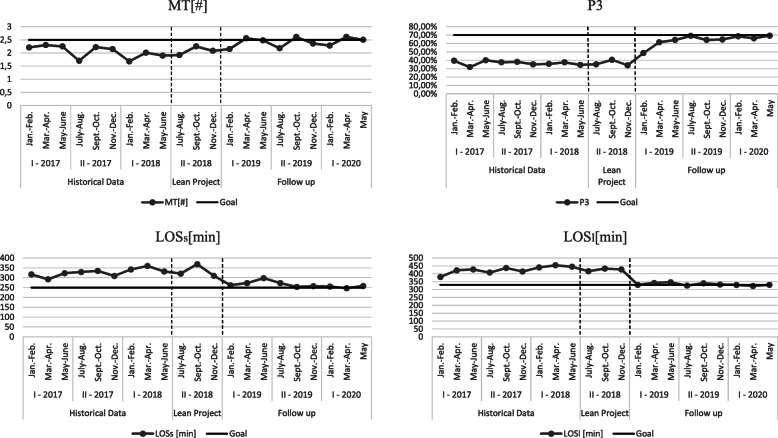
Run charts

Table 2 and Fig. 4 shows the results achieved through the implementation of the pilot project. The objectives were not reached for all indicators; however, the results improved over time.

**Fig. 5:**
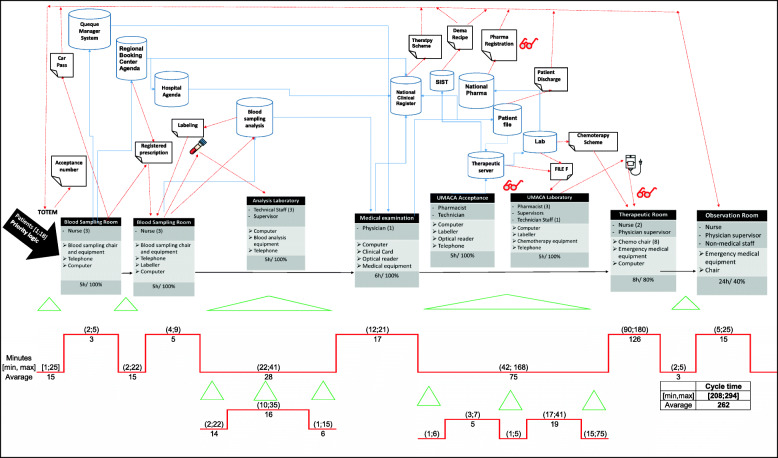
To-be process represented by VSM

Figure 5 shows the to-be state of the same process analysed in Fig. 3. From the cycle time analysis of each process step, the areas of waste eliminated are clear.

The incremental improvements in process performance over time are explained by the need for staff to learn new procedures in the early period. In addition, the patients’ resistance to changing their habits also slowed down the improvement in performance. Patients have been educated over time, through an intense communication activity based on visual management systems and telephone reminders.

In addition to the results showed in Table 2, the pilot project had a positive impact on the performance of other patient pathways in the medical department. The cycle time variability reduction and the levelling of the service demand allowed the UMACA and the analysis laboratory to better plan their activities. The new layout reduced waste due to unnecessary movement. Nurses walk 2 km less per day and doctors 1.5 km less per day. Software integration has reduced data logging time by 35 min per day for each doctor. Patients have evaluated the change positively. In particular, they have experienced a drastic reduction in wait times, and greater attention from the medical staff. Increased privacy and a precise time of service are other improvements reported by patients.

Finally, the clinical staff was satisfied with the new procedures because they reduce overloads and allowed for better planning of activities. They say that dividing patients into time slots based on clinical priority reduces stress and simplifies the coordination of activities with other departments. The success of the project was communicated internally and externally to the organization. In June 2019 the results were celebrated with a formal team award ceremony. The resulting Report A3 was posted on the bulletin boards in the hospital wards and in the reception area. By means of an internal circular sent to all medical directors, the directorate officially thanked the members of the lean team and highlighted the excellent results achieved in terms of waiting lists and process time reduction. In addition, the directorate funded the lean team’s participation to national conferences in order for the team to discuss the project. The improvement activities and results were described and summarized in an official report sent to the regional health authority and cancer patient associations (the latter were also given an evaluation form and an invitation to observe the optimised process in the field). Reporting was carried out by the hospital directorate and the project manager.

#### Lean dissemination and adoption in the hospital

**Fig. 6:**
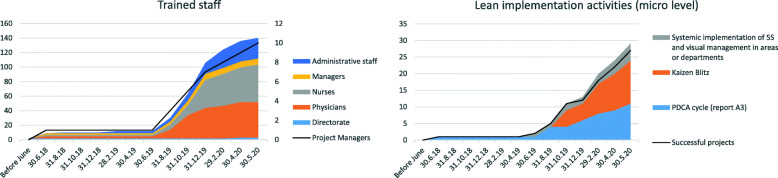
Lean projects and dissemination activities

Following several meetings between the directors and the primary doctors of the medical area, it became clear that there was a willingness to implement further improvement projects in other medical wards. The feedback from the pilot project team was a strong convincing factor. Moreover, the results of the external communication of the pilot project played a critical role in increasing the desire for emulation. The regional authorities requested for the project team to co-design the diagnostic and therapeutic care pathways (PDTA) of the haematology patient pathway inside the regional network. The patient association lobbied for similar projects to be implemented in other clinical oncology pathways. The change of internal context and enabling factors were of great importance at this stage. The drive to disseminate lean was characterized by both the need to improve process performance and to the desire to emulate the success of the project pilot. In addition, increased trust in the lean method has prompted the directors to provide a peer internal training program in the medical area. In June 2019, members of the pilot project lean team were promoted to the position of lean champions. Their role was to disseminate the lean methodology in the medical area and to train colleagues. The hospital directorate set up the Lean Support Office and assigned to it the three non-clinical resources that had already supported the pilot project. The first methodology to spread throughout the medical department was 5S. According to the lean sponsors, this methodology was a prerequisite for implementing lean methodologies in all wards and for facilitating inter-process lean implementation. Visual management systems have been implemented to facilitate changes and standardization of activities and to guide the patient through the hospital. The 5S methodology and visual management, which was initially underestimated by the medical staff, has solved many problems in the working environment. Increasing the availability of tools, simplifying the transmission of documentation, reducing errors in medical records and nursing diaries, reducing the duplication of requests and medical documentation, creating flexible workplaces, less movement and transportation in the hospital, and increasing patient autonomy are just some of the improvements achieved. However, the most important result to be achieved was an improvement in workplace wellbeing. Among the most used tools for 5S implementation and visual management are: checklists, one point lessons, kaizen forms, horizontal and vertical marking, red tags, Kanban, spaghetti charts. Finally, the demand map was implemented to trace the patient flow across the departments of the medical area and the vertical swim lanes and the resources/process matrix were utilized to identify staff involved in several processes and the potential bottlenecks (in addition to the UMACA and the blood chemistry laboratory). As of August 2019 many other lean projects have launched sometimes spontaneously and sometimes at the demand of department heads or project managers (Fig. 6). In August 2019, three projects were undertaken in the medical oncology for thoracic pathology and the medical oncology wards. Two of them concerned the same clinical pathway addressed in the pilot project, and the last one was the harmonization of protocols for caring for an oncological patient between departments. Each project has been implemented following the PDCA cycle (using the A3 report framework) with the support of one of the lean champions, who was assigned the role of project manager. Teams of three doctors and one nurse were dedicated to each project. In the planning phase, the tools adopted in each project were: spaghetti charts, VSM, Gemba Walk, standardized data collection sheets (both for patients and physicians), control charts, 5 Why or alternatively the Fishbone Diagram, definition of SMART objectives. In the “Do” phase, the solutions adopted for the resolution of problems are derived from Just in Time and agile approaches (especially for software’s’ integrations management). The pilot project A3 report was used as a knowledge management tool and resulted to be of great value to guide the implementation of the three projects. The members of the pilot project team supported their colleagues during the implementation of the three projects. This resulted in a positive impact on the quality and timing of the data collection activities, the drafting of the VSM, the definition of the KPIs and especially the root cause analysis. Even though the negotiation was simplified by peer training, support from more experienced colleagues and project management by a doctor, organizational and structural barriers emerged. The difficulty in getting the new procedures accepted, the impossibility of optimizing the layouts and the “not always respecting” the authority of the project manager limited the performance improvement. Although not all potential solutions have been implemented, the results obtained are evidence of the success of the projects.

In September 2019 the diagnostic department started 5S and visual management implementation initiatives. In October 2019 the same initiatives were undertaken in the surgical department. These initiatives were spontaneously implemented. The managers of these departments have asked the hospital director to introduce lean in their departments. Given the maturity of the method and the number of doctors trained, hospital managers did not consider it possible to undertake systemic improvement pathways in all departments. However, they have changed the organizational structures of the departments into matrix structures. Two doctors with lean experience, per department, have been assigned the role of project manager. The project managers have sponsored peer training and Kaizen blitz activities throughout the hospital departments. In the period October to December 2019 more than 60 doctors and nurses were trained in 40-h courses by their colleagues (Fig. 6). Three Kaizen blitz projects in the diagnostic department and two Kaizen blitz projects in the surgical area were carried out (Fig. 6). In addition, a PDCA cycle project was implemented in the medical area for the stocking and tracking of drugs and instruments. Moreover, the two bin Kanban systems, drug tracking tools, optimisation of the position in the storage layout and systems for the analysis of consumption time series were implemented.

In December 2019, in all the departments discussed so far, doctors were involved in continuous improvement activities, with projects structured through the use of both PDCA cycle and Kaizen blitz. The activities were undertaken spontaneously without the supervision of a manager and without any impact on daily clinical activity. The maturity of the methodology, the support of colleagues, and trust were enabling elements. However, some barriers such as infrastructural constraints and coordination of doctors and nurses and information systems have frequently affected the implementation of the method and two projects failed.

Due to the success of implementations at the micro level, managers have attempted to implement the lean methodology at the meso level. Hospital managers discussed, formalized and communicate in organization the Lean Strategic Plan. In January 2020, the Lean Support Office was transformed into a lean projects control room and renamed as the Operations Management Office. The role of this office is to define lean development policies and to supervise continuous improvement activities. The office has been placed in line with the strategic direction. Two lean project managers, two hospital managers, and three administrative officers have been assigned to it. Lean assessment, to evaluate the degree of lean maturity in organization, and Honshi Kanri, to strategically govern change activities, were implemented to the organizational level. While the lean assessment revealed an increase in both advance in the use of lean tools and the principles behind them, the governance of strategic implementation through Honshi Kanri did not seem to provide the foreseen results. Operations management office project managers did not always agree with hospital directorate on project prioritization. In addition, there often were disagreements between the Operations Management Office staff and departmental project managers about when to launch a project and how to manage it and communicate project results. Although there were many process improvement projects underway, these have not always been decided harmoniously between the Operation Management Office and the hospital departments. Moreover, many projects undertaken spontaneously by lean teams were not communicated to the Operations Management Office, which was therefore unable to govern the dissemination of the method. Medical leadership in departments seemed to dominate over managerial leadership; thus, there is great difficulty in strategically governing continuous improvement.

**Fig. 7:**
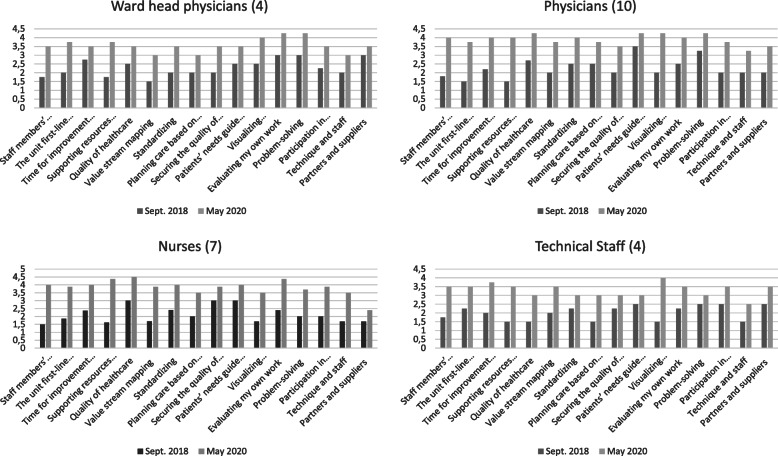
Responses to the closed questions of the questionnaire

The marked differences in the responses to the closed questions of the questionnaires submitted provide significant evidence of how lean has spread throughout the organization (Fig. 7).

The marked differences in the responses to the closed questions of the questionnaires presented provide significant evidence of how lean has spread throughout the organization. In particular, the results show how standardisation, self-assessment, time for improvement and peer-to-peer training have become part of everyday working practice. Furthermore, problem solving and collaborative decision-making show significant improvements. These improvements were witnessed not only by management but also by doctors, nurses and technical staff in the medical area.

After the pilot project and the initial push for implementation by management, internal contextual factors changed radically within the organization. While initially sponsorship and management involvement were necessary for lean implementation, today the methodology is independently disseminated. In particular, small improvement groups have emerged that are able to address various challenges. Process vision and patient focus have become part of the hospital culture. Doctors claim that continuous improvements simplify daily work, save time, and increase the level of service and the number of services provided. However, although these changes occurred at the micro level, the organization failed to direct change at the strategic level. Thus, harmonization of lean projects according to the strategic direction of the facility has yet to be achieved.

## Discussion

In accordance with the findings of many researchers [10, 16], this case study showed how a careful, context-driven lean introduction strategy facilitated the dissemination of lean - at micro level - within the hospital. The decision to implement lean was precipitated by external factors, including the need to improve the performance of processes in the medical area and to follow the example of other successful hospitals. The in-depth training by an external specialist and the pilot project, characterized by interdepartmental activities, the need for a systemic approach based on the Deming Cycle and the constant support of the external consultant, allowed the participants to acquire the necessary skills to support - sufficiently - the lean implementation in the clinical pathways of the medical department and to train their colleagues. The results of this project have been manifold. At the process level, there was a significant reduction in the patients’ length of stay, the wait times for haematological patients, the process time variability, and an increase in the number of daily chemotherapy therapies performed. At the medical area level, a spontaneous spread of the culture of improvement has emerged. Directorate commitment, motivation of the medical department staff and management, and the presence of a consultant were the main enabling factors for the success of the pilot project. In turn, the results of the pilot project were the trigger for the spread of lean in the hospital. The pilot project itself, and the changes made to standard procedures that were inspired by the intervention, altered the contextual elements, mirroring the MUSIQ model [18, 26, 36]. Moreover, as trust and maturity raised, the speed of lean dissemination increased. This confirms that knowledge of the lean method tends to reduce organizational barriers and resistance [5, 21, 51, 52]. Kata training and coaching were other key elements for the dissemination of the methodology. Initially, the consultant carried out the training activity, and after the pilot project, the team members became trainers and project managers; in this way, lean spread in the organization spontaneously. Moreover, as stated by many researchers [12, 21, 46], the matrix structure and project managers helped the staff to support and better coordinate process improvement. The many projects activated in the period July 2019–March 2020 are the measure of the diffusion itself.

However, some issues have arisen. For the new working procedures, the willingness of and the acceptance by the staff is crucial to achieving and sustaining the results of lean initiatives; where this did not occur, conflicts arose and the speed of change slowed. In addition, although in the early stages of implementation the bottom-up approach must prevail over a top-down approach to facilitate consensus and trust among physicians, nurses, and all workers, during the dissemination phase a greater equilibrium between the two decision-making approaches must be achieved. In accordance with [2, 5, 10], this case study demonstrates the importance of the right balance between bottom-up and top-down approaches. Medical leadership tends to dominate managerial leadership such that continuous improvement, even though it takes place in clinical processes, does not follow the strategic organizational guidelines. This leads to conflicts between managers and medical staff. Organizational, technical and infrastructural obstacles have hindered the adoption of the methodology. It is clear from what has been found that the introduction strategy was correct, but that the implementation at the strategic level has not yet taken place. The context has changed considerably from an organizational point of view, but some barriers have not been overcome. The management, which strongly sponsored and supported the introduction and implementation of lean, was subsequently unable to guide the implementation at the strategic level.

Our adaptation to the MUSIQ model is useful for interpreting the relationship between lean introduction strategies and changing contextual elements. Looking backward through this model allows us to understand the links between contextual elements, lean implementation and outcomes.

## Conclusions

This study revealed that the strategy of introducing lean has improved readiness, sustainability and confidence in the method within the organization. The growing maturity of the organization has encouraged lean dissemination. However, the choice of strategy depends heavily on contextual factors. The two factors, therefore, influence each other. Although the introduction strategy may facilitate the introduction of lean, it may be less important when certain organizational, technical and infrastructural barriers remain. This is particularly relevant for systemic implementation. Contextual elements, which changed over time, influenced the success of the implementation at micro-level. At the meso-level, however, the organization has not reached the maturity for a systemic implementation of the method.

As has already been shown in the literature, the determining factors for introducing the methodology refer to external and internal pressures. The level of commitment of both the leadership and management are decisive for the success of the implementation only if the staff is motivated. Furthermore, the analysis shows that managing lean implementation at the micro and meso-levels requires different types of efforts. While the level of maturity speeds up the adoption of lean at the clinical level, it is not true that the dissemination of lean at the operational level inevitably translates into its application at the strategic level. Medical leadership, reinforced by the success of lean project implementations, could instead undermine proper implementation at the meso-level. This experience strengthens the MUSIQ model and complements it by showing the importance of the lean introduction strategy and its impact on contextual factors.

### Limitations and future research

The main limitations concern the complexity of detecting and analysing all the relevant social and organizational aspects that have characterized the introduction and dissemination phases and the observation period of the dissemination phase. Moreover, the expert content analysis could introduce opportunities for misinterpretation of the data. The relationship between the contextual elements and the pilot project results were mainly assessed through participant and patient reports, document studies, and observations. The authors used data triangulation and a review of hospital staff to overcome the limits of the content analysis. Given the specificity of the hospital’s contextual factors and strategic choices, it is also clear that the case study cannot be generalized.

The sustainability aspect of lean was not considered because the observational study was conducted over a period of only 2 years. To understand this issue, the authors will investigate the socio-technical aspects of lean and how the context supports continuous improvement over time.

## Supplementary Information

**Additional file 1.**

## Data Availability

The datasets used and/or analysed during the current study available from the corresponding author on reasonable request.

## Abbreviations

HS: Hospital Setting
IT: Information Technology
LOS: Length of stay
MT: Number of chemotherapies per chemo chair
MTM: Methods-Time Measurement
MUSIQ: Model for Understanding Success in Quality
OECI: Organization of European Cancer Institutes
P3: Patients undergoing chemotherapeutic infusion within three hours of hospital admission
PDCA: Plan-Do-Check-Act
UMACA: Antiblastic Chemotherapy Handling Unit
VSM: Value Stream Mapping
