# Supplementary material for: Lean adoption in hospitals: the role of contextual factors and introduction strategy
Source: BMC Health Serv Res. 2021 Aug 28;21:889. doi: 10.1186/s12913-021-06885-4 (PMC8403367; doi:10.1186/s12913-021-06885-4)
Supplement: Supplementary file 1 — Additional file 1. [file 12913_2021_6885_MOESM1_ESM.docx]

**The Lean in Healthcare Questionnaire (LiHcQ)**

**The questions below are intended to provide a description of Lean and to what extent Lean occurs at your workplace.**

Please complete the questionnaire by **marking the response alternative that best describes your workplace.** Statement 1 means that the unit has nothing of what that statement implies; statements 2-5 mean that what the statement concerns is present at your unit and that the maturity is gradually increasing.

First, you are to take a stand on statement 1, if the statement is not applicable to your unit, you need to consider statement 2, etc. If you consider that, e.g., statement 4 is applicable to your unit, then statements 2 and 3 should be applicable as well; to answer the question, mark only statement 4.

**1. Staff members’ commitment to Lean**

(Words marked with a * are explained after the question. All descriptions provided below have the same meaning throughout the questionnaire. If a word marked with a * appears in another question, you can go back to the earlier description).

| ( ) 1. | Staff on the unit show no commitment to or have a negative attitude toward Lean*. |
| --- | --- |
| ( ) 2. | Staff members see Lean as a temporary project and are only willing to devote limited time and commitment to improvement work**. |
| ( ) 3. | The staff support Lean. They devote time and effort to coming up with ideas for improvement, but not concerning how to transform these ideas into new ways of working or solving problems. |
| ( ) 4. | Staff members are a driving force for making Lean part of their daily work. They want to develop their work based on the ideas for improvement and have long-term solutions to problems. |
| ( ) 5. | Everyone knows that staff efforts are essential to Lean. Improvement work is considered an important part of the daily work, where the focus is on both finding new solutions and retaining earlier solutions. |

**Common to Lean is that staff members focus on continuous improvements. Having flow means that healthcare is provided in a coherent, uninterrupted chain. The quality of care provision and the patient’s desires are in focus.*

***Improvement work means that staff on the unit solve problems and improve, for example, working methods, chains of care, flow and the quality of care provision. Evaluating working methods and adopted improvements is always part of improvement work.*

**2. The unit first-line manager’s commitment to Lean**

(If you are a first-line manager state yourself)

| ( ) 1. | The unit first-line manager shows no commitment at all or has a negative attitude toward Lean*. |
| --- | --- |
| ( ) 2. | The unit first-line manager sees Lean as a temporary project and is only willing to devote limited time and resources to improvement work. |
| ( ) 3. | The first-line manager supports Lean and approves of time and resources being devoted to improvement work. However, he/she does not work actively to make Lean part of the daily work, seeing it mainly as something staff should do. |
| ( ) 4. | The first-line manager asks questions using a coaching style concerning problems and suggestions for improvement. He/she is a driving force for making Lean part of the daily work. |
| ( ) 5. | Everyone feels the first-line manager is important to Lean. The first-line manager uses a coaching style, which has helped staff come up with new solutions that mean improvements for them and/or patients. There is equal focus on finding new solutions and retaining previously solutions. |

**3. Time for improvement work**

| ( ) 1. | No time is specifically allocated to improvement work. |
| --- | --- |
| ( ) 2. | There is rarely time allocated to improvement work. |
| ( ) 3. | Most of the teams on the unit have frequent improvement meetings or similar. The teams have made different progress concerning how they carry out improvement work. |
| ( ) 4. | All teams on the unit have frequent improvement meetings or similar, which means that all staff are involved. |
| ( ) 5. | Improvement work is part of the regular work, and every one feels that improvement work is important to the quality of healthcare provision. |

** A team consists of staff with different functions; there may be two or more persons from different professions. But the teams may also consist of staff from the same profession if they contribute different knowledge.*

**4. Supporting resources for work with Lean**

| ( ) 1. | No person/persons on the unit have been appointed to support the staff in making Lean part of the regular work. |
| --- | --- |
| ( ) 2. | A person/persons on the unit have been appointed to support the staff in making Lean part of the regular work. |
| ( ) 3. | The person/persons who are supporting the staff have received the training needed for the task. |
| ( ) 4. | The appointed person/persons work closely with the staff and are a driving force for improvement work. |
| ( ) 5. | Everyone feels that the person/persons who support the staff are important, have great competence and provide good support for the teams. |

**5. Quality of healthcare**

| ( ) 1. | No one on the unit makes any effort to understand what the patients consider valuable*. |
| --- | --- |
| ( ) 2. | The staff on the unit have begun evaluating what the patients consider valuable in an unstructured way. The teams have progressed differently concerning how to collect this information. |
| ( ) 3. | What the patients consider valuable and what promotes and does not promote this is frequently discussed in most of the teams. |
| ( ) 4. | Most staff can see and describe what happens on the unit and what they personally contribute that creates and does not create value for the patient. |
| ( ) 5. | Everyone on the unit has a new way of thinking and is highly skilled in identifying what the patients consider valuable. What the patients consider valuable is evaluated continuously. |

**What the patients consider valuable is efforts that improve the quality of healthcare or address the patient’s desires. What the patients do not consider valuable is e.g. waiting, not having any influence, not receiving information or when the staff need to search for material.*

**6. Value stream mapping**

| ( ) 1. | On the unit there is no value stream mapping* aimed at increasing the quality of care provision or describing what the patients consider or do not consider valuable. |
| --- | --- |
| ( ) 2. | Some or parts of the chains of care on the unit have been mapped. The quality of the value stream mapping varies and the mappings are sometimes out of date. Through recurrent problems on the unit, different kinds of waste** have been identified. |
| ( ) 3. | Waste has been discovered using information on what patients consider valuable and what can increase the quality of the care. The most common chains of care on the unit are mapped; some mappings are detailed others are not. |
| ( ) 4. | On the unit there are updated and detailed value stream mappings of the chains of care. The mappings are updated at least once a year and are placed visibly at places where the staff need the information. |
| ( ) 5. | On the unit there is a well-developed structure for performing value stream mappings and using the information from them. The mappings are updated continually and are used to improve care provision. |

**Performing value stream mapping of the chain of care means that the staff map in detail the patients’ care, e.g. document how long the patient had face-to-face contact with the healthcare staff, how long the staff or patients had to wait, what tasks have been conducted, by whom and how long it took. The aim of value stream mapping is to gather information on what can be improved by reducing waste and increasing the quality of care provision.*

***Waste can include: waiting time for both staff and patients, patients not receiving understandable information or staff searching for material. Searching for material or finding short-term solutions are examples of waste that show that the root cause to a problem has not been solved. Detecting waste can occur, for instance, when staff notice that material is always missing from the storeroom.*

**7. Standardizing**

| ( ) 1. | The unit uses no routines* when carrying out the work. |
| --- | --- |
| ( ) 2. | Some teams on the unit have begun creating and using routines. However, these are not always written down or may be in the form of a basic checklist. |
| ( ) 3. | Some teams have routines. The routines have begun to be clearer, more detailed and are written down. |
| ( ) 4. | Most teams have routines. The teams have also begun investigating whether the chosen routines are always used. |
| ( ) 5. | Everyone on the unit is highly skilled at developing and following routines. When errors and problems are found, the routine is used to identify the root cause of the error/problem. Questions asked are: Was the routine followed? Does the routine need to be clearer? Is there a need for more knowledge? |

**In this questionnaire, the term routines refers to the working methods the unit staff themselves have decided to use. This may involve calibrating equipment on certain days or taking the same samples in response to specific symptoms. The above statements include only routines the unit has created based on, e.g., the national guidelines.*

**8. Planning care based on patients’ needs**

| ( ) 1. | On the unit, changes in patient flow as well as the resources required (e.g. time, staff or competence) are largely unforeseen. The unit staff do not plan in advance*. |
| --- | --- |
| ( ) 2. | Staff on the unit have begun examining changes in patient flow and which resources (e.g. time, staff or competence) are required. |
| ( ) 3. | The unit staff plan when different resources are to be used based on patient flow statistics. This may mean, e.g., higher staffing levels on days when the patient flow is higher. |
| ( ) 4. | The unit staff make early contact with their patients to more precisely predict which resources are needed. This may mean offering patients a health control. |
| ( ) 5. | Everyone on the unit is innovative and highly skilled in proactive planning. To achieve good flow in the chain of care** the unit staff use different ways of involving*** patients in their own healthcare. |

** Planning ahead to even out the workload involves unit staff trying to estimate patients’ needs in advance. This may mean considering the fact that more patients seek care on a certain day or that many want vaccinations during a certain period.*

*** To have good flow in the chain of care means that the chain is coherent and of suitable quality. In practice, this means that the staff aim to reduce waiting times for patients and themselves and that the appropriate staff member provides the right care at the right time. Different resources, e.g., information or material, shall be accessible and visible for staff where needed. A chain of care can start when the patient contacts the healthcare system and staff schedule the patient, when the patient is welcomed and registered, the visit itself, etc.*

**** To create flow in the chain of care, the patients can be involved by, e.g., encouraging them to seek advice on healthcare on the web to a larger extent, letting the patient do some preparations at home or answering questions at home before the visit.*

**9. Securing the quality of healthcare through automatic quality controls***

| ( ) 1. | Staff on the unit feel that quality controls are time consuming or do not know how they can be performed automatically. It may even be the case that staff have low awareness of quality of care in general. |
| --- | --- |
| ( ) 2. | Staff on the unit have begun investigating how automatic quality controls can be performed to evaluate the quality of care provision and working methods. This approach is, however, unstructured and only used by a few teams. |
| ( ) 3. | Some teams have a structured approach regarding investigating how automatic quality controls can be implemented and used. |
| ( ) 4. | Almost everyone on the unit is involved in making quality controls occur automatically. Each individual staff member strives to offer care of the right quality by preventing in different ways errors from occurring and evaluating the quality of their own work tasks. |
| ( ) 5. | Everyone at the unit is highly skilled regarding implementing and using automatic quality controls. The work tasks are developed to ensure that right quality of care is provided from the outset and that a minimum of time is spent on quality control. |

**Automatic quality controls may involve having to document important information in the patient file in order to be able to continue documentation. It may also include that equipment cannot be used in the wrong way or that the plugs are made so they can only fit in one way.*

**10. Patients’ needs guide healthcare work**

| ( ) 1. | On the unit, it is not what the patients desire* that guides the healthcare provided. |
| --- | --- |
| ( ) 2. | The teams have begun basing their work on what the patient’s desire. Executing work tasks that are not desired is still common; this can involve preparing for a patient or an examination hours in advance or without prescription. |
| ( ) 3. | Most teams base their work on what the patients desire, and to achieve good flow in the chain of care the teams use different signs and signals**. The staff have begun helping each other create good flow on the entire unit. |
| ( ) 4. | All teams provide care based on what the patients desire, though their progress is different. Knowing in advance what is desired facilitates good flow in the chain of care. The staff know what has occurred/will occur before/after their work, and they use signs and signals to show when the next staff member should offer care. Adopted improvements are sustained. |
| ( ) 5. | Staff on the unit have a well-developed approach to basing their work on patients’ desires. There is continuous development to improve signs and signals so as to create good flow in the chains of care. |

**Basing care provision on patients’ desires may include more staff being on duty when patient demands are high, e.g. a specific day of the week. It may also include changing opening hours based on patients’ desires.*

***Visible signs or signals can be color marks, text, sounds or signs, e.g., on the computer, notes or a white board. Signs/signals can show, e.g., that XX is ready to be signed, that the patient has arrived or that the doctor has seen the patient and that the Licensed Practical Nurse (LPN) can perform his/her work task. Signs or signals can also show where material, equipment or staff are located or whether something is about to run out of stock or which improvements, errors and problems are present on the unit.*

**11. Visualizing improvements so that everyone can work based on them**

| ( ) 1. | Improvements on the unit are not visualized*. |
| --- | --- |
| ( ) 2. | Parts of the unit visualize some improvements, however, in an unstructured manner. The staff want to learn and know more about how improvements can be visualized. |
| ( ) 3. | Improvements are visualized and placed where the staff need them on almost the entire unit. Descriptions of the improvements are updated, and the staff are testing what needs to be visualized. |
| ( ) 4. | Improvements are visualized on the entire unit and placed where the staff need them. Visualized improvements are updated and concern different matters. |
| ( ) 5. | Everyone on the unit has fresh ideas and is highly skilled in visualizing improvements. These improvements are updated and their placement is well thought through. |

**Visualizing improvements may involve staff putting up photos of what material they have decided to have in a treatment room or making visible a number of mistakes, discrepancies and measures taken on the unit. It may also involve descriptions of new working methods, evaluations and further developments of working methods or how the staff have solved the problems by dealing with root causes.*

**12. Evaluating my own work**

| ( ) 1. | It is common for the first-line manager or senior management on the unit to make decisions concerning evaluations* of healthcare, and it is difficult for staff to influence how these evaluations are designed and executed. |
| --- | --- |
| ( ) 2. | Some staff members have begun on their own looking for different methods to evaluate their own work and various chains of care. |
| ( ) 3. | Staff have begun, on their own initiative, evaluating some work tasks and chains of care; some have also designed different approaches to performing the evaluations. |
| ( ) 4. | The evaluations are performed to various extents on the unit. The evaluations follow up performed work and most of the chains of care, but not individual staff members. |
| ( ) 5. | Everyone on the unit is highly skilled at evaluating the performed work and chains of care. The staff both develop and use new ways of evaluating improvements. |

** Evaluating implies measuring and following up a chain of care or other efforts, e.g. changed opening hours or a new working method should always be evaluated to determine whether the new standard should be made permanent. When performing evaluations, the staff can measure, e.g., the patient flow, patient satisfaction or how long it takes for the patient to get well.*

**13. Problem-solving**

| ( ) 1. | Improvement work is unplanned and unstructured. Staff often focus on the concrete problem and not on its root causes. Errors and problems are solved by “putting out fires”. |
| --- | --- |
| ( ) 2. | Improvement work on the unit is more structured and staff have started using different methods to solve problems*. They search for the root causes of errors and problems. |
| ( ) 3. | The staff are skilled in problem-solving; they routinely solve errors and problems in different ways. |
| ( ) 4. | The staff take an experimental approach to improvement work. They always evaluate improvements and make adjustments based on evaluation results. |
| ( ) 5. | There is a well-developed structure for improvement work on the unit. Improvements are conducted to solve problems and to further develop flow, the chain of care and working methods. |

**Using different methods or tools for problem-solving may mean that the staff investigate the root causes of errors and problems, that they evaluate their working methods or that the involved staff don’t use second-hand information, but instead go and see for themselves and investigate the problem.*

**14. Participation in decision-making**

| ( ) 1. | Decisions are made by the first-line manager without communicating with the staff. |
| --- | --- |
| ( ) 2. | Sometimes the staff participate in information meetings about important decisions and changes, but most often it is the first-line manager who makes decisions without communicating with the staff. |
| ( ) 3. | The first-line manager and staff have structured meetings when decisions are made. The first-line manager is interested in and sometimes takes into consideration staff members’ opinions. |
| ( ) 4. | Structured meetings are held on the unit, and the first-line manager aims at consensus. |
| ( ) 5. | On the unit, there is a well-developed structure when decisions are to be made. The staff participate, they are committed and decisions are made by consensus. Different alternatives are always considered. |

**15. Technique and staff**

| ( ) 1. | The staff are not involved when purchasing products. |
| --- | --- |
| ( ) 2. | The first-line manager has begun consulting involved staff when purchasing products. |
| ( ) 3. | Some staff are involved when products are purchased. Products purchased should improve the flow, increase the quality of care provision or support staff in their work. |
| ( ) 4. | Involved staff are always engaged and participate when new products are purchased. |
| ( ) 5. | The unit has a well-developed structure for purchasing and implementing new products. The products are always tested and evaluated by involved staff prior to implementation. |

**16. Partners and suppliers**

| ( ) 1. | The staff have no contact with partners* and suppliers*. |
| --- | --- |
| ( ) 2. | Some staff on the unit communicate with partners and suppliers concerning orders or related topics. |
| ( ) 3. | Some staff at the unit have continuous communication with partners and suppliers and negotiate with them concerning, e.g., prices, delivery times or waiting times. |
| ( ) 4. | Several staff on the unit negotiate with partners and suppliers and support them in ensuring that their services, e.g., fit into the flow on the unit, maintain a high quality or reduce delivery costs. |
| ( ) 5. | On the unit there are well-developed collaborations and a structure for negotiating with partners and suppliers and supporting them. |

** Partners and suppliers may include the pharmacy, other healthcare institutions, purchased services such as cleaning and janitor duties, and those who deliver various goods.*

**First stage: Open-ended questions**

*Please answer the questions below. The results will be summarized and discussed during a meeting session.*

1) Why did the organization commit to the Lean implementation process?

2) What was your department's feedback on the decision to implement a continuous improvement model?

3) What do you believe are the enabling or failing factors affecting lean implementation in your department?

**Second stage: Open-ended questions**

*Please answer the questions below. The results will be summarized and discussed during a meeting session.*

1) Do you perceive that the enabling and critical success factors that influence lean implementation have changed in your department? Please explain your answer.

2) Do you agree that the perception of Lean methodology in your department has changed? Please explain your answer.

3) Do you think lean is spreading across the organization? Please explain your answer.
